# Depressive symptoms among orphans and vulnerable adolescents in childcare homes in Nepal: a cross-sectional study

**DOI:** 10.1186/s12888-020-02863-y

**Published:** 2020-09-25

**Authors:** Kumari Bandana Bhatt, Tawatchai Apidechkul, Peeradone Srichan, Navin Bhatt

**Affiliations:** 1 Shool of Health Science, Mae Fah Luang University, Chiang Rai, Thailand; 2 Department of Health Services, Ministry of Health and Population, Kathmandu, Nepal; 3 Center of Excellence for the Hill Tribe Health Research, Mae Fah Luang University, Chiang Rai, Thailand; 4 Bayalpata Hospital (Nyaya Health Nepal / Possible), Achham, Sanfebagar, Nepal

**Keywords:** Depressive symptoms, Orphans and vulnerable adolescent, Child care homes, Presence, Factor associated

## Abstract

**Background:**

Orphans and vulnerable adolescents (OVAs) living in child care homes (CCHs) are vulnerable to depressive symptoms due to a poor environment and a lack of receiving good care and love from their parents. This study aimed to estimate the presence of depressive symptoms and determine factors associated with it among OVAs living in CCHs in Nepal.

**Methods:**

A cross-sectional study was conducted to collect the information from OVAs aged 13–17 years living in 22 CCHs from five districts of Nepal. The CCHs were selected by a simple random method. A validated questionnaire and the Beck Depression Inventory-II (BDI-II) were used to assess depressive symptoms among the participants. Those with mild to severe BDI-II scores were defined as having clinically relevant depressive symptoms. Logistic regression was used to detect associations between variables at the significance level α = 0.05.

**Results:**

A total of 602 adolescents participated in the study; 51.0% were females, the average age was 14.7 years, and 32.2% were members of indigenous groups. The overall presence of clinically relevant depressive symptoms was 33.2%. After controlling for all potential confounding factors, five factors were found to be associated with depress among OVAs. Females were 1.96 times more likely to develop depressive symptoms than males (95% CI = 1.36–2.83). Those adolescents who used alcohol were 3.42 times more likely to develop depressive symptoms than those who did not (95% CI = 1.16–10.12). Those who had health problems were 2.00 times more likely to develop depressive symptoms than those who did not (95% CI = 1.36–2.94). Those who had low social support were 1.81 times more likely to develop depressive symptoms than those who had high social support (95% CI = 1.08–3.03), and those who had been bullied were 1.97 times more likely to develop depressive symptoms than those who were not bullied (95% CI = 1.23–3.15).

**Conclusion:**

The magnitude of clinically relevant depressive symptoms in adolescents living in CCHs was found to be high in Nepal. There is an urgent need for effective intervention to curtail this problem among OVAs in CCHs in Nepal, with a focus on females, alcohol users, those with physical health problems and with less social support, and those who are bullied.

## Background

Adolescence is an important period of growth and development [1]. Family and guardians play an essential role in the health and illness of their children [1]. The growth and development of adolescents are also affected by the environment, especially when they have experienced the loss of parents [2]. A supportive environment in the family, school, and community is important for adolescents to develop and maintain positive social and emotional habits [3]. Orphans and vulnerable adolescents (OVAs) living in child care homes (CCHs) are at high risk of physical, sexual, and emotional abuse or exploitation and face social discrimination and stigma, which are serious challenges in developing countries [4]. These problems in this core group of adolescents result from a lack of care and support during the grieving process and an inadequate environment without parents, which can significantly increase the risk of mental and behavioral disorders, particularly depression [5].

Depression is defined as a major mental health problem among adolescents [6]; the global burden of depression had increased by 18.4% from 2005 to 2015 [7]. Depression affects more than 264 million people, equivalent to 4.4% of the world’s population [8]. The prevalence of depression is reportedly high among people living in low- and middle-income countries [9]. Approximately 27.0% of all depressed people live in the Southeast Asia region, which includes Nepal. Nepal has reported a 3.2–4.0% prevalence of depression [8]. Depression is the most persistent and preventable mental health problem in adolescents [10], and a high prevalence of depression has been reported for adolescents living in CCHs in Nepal [11].

Previous studies have shown that the prevalence of depression among orphans ranges from 20.0% [5] to 68.0% [12]. The World Health Organization (WHO) reported that depression is the leading cause of illness and disability [3]. Moreover, depressed people are twice as likely to commit suicide and to die prematurely compared to those who do not have depression [13]. Suicide is the third leading cause of death in the population aged 15–19 years and particularly impacts those who have depression [14]. Major depression in adolescents impairs their performance at school or work and hinders interactions with their friends and family [3]. It affects their physical and social life and impinges on their human rights, economic, educational, productive, cultural, and reproductive rights and has a notable negative impact on the quality of life, especially in adolescents aged 15–17 years [13, 15].

CCHs are intended to be caring and safe living environments for adolescents who have no one to support their life [16]. In Nepal, it is very common for adolescents to live in CCHs owing to difficulties in their life such as the loss of their parents or if their family does not want or is unable to care for them [16]. In CCHs, adolescents face many problems such as mistrust, maltreatment, insecurity, neglect, risk of abuse, and exploitation, which significantly increase the risk of emotional and behavioral problems [17]. Lack of care and support during the grieving process of their parents’ death, an inadequate environment with poor living conditions, and fear about school and the future in the absence of parents may lead to depression [5]. Adolescents in CCHs are also known as orphans and vulnerable adolescents (OVAs). OVAs typically have a low socioeconomic status, which is very challenging in developing countries, particularly in Nepal [4]. There are more than 400 million orphans globally [18]. In Nepal, it was estimated that were more than one million orphans in 2018 [19]. Nepal is a developing and economically poor country that has undergone long-term social, political, and civil conflict that has substantially increased the number of adolescents living in CCHs in the country [20]. The government of Nepal operates several programs to address this issue; however, depression is still reported as a major public health and social problem [16].

The Ministry of Health and Population (MOHP) of Nepal is responsible for delivering quality health care services to all Nepalese people. Universal health coverage was one of the priority agenda items of the National Health Policy 2014 and is stated in the National Health Sector Strategy 2015–2020 guide for the overall health plan in Nepal [21]. In 1996, Nepal’s mental health policy was formulated to ensure the availability and accessibility of minimum mental health services for the population [22]. In 2018, one specialized mental hospital and 18 outpatient mental health facilities were available, none of which are reserved for adolescents. Less than 1 % of health care expenditure has been allocated for mental health by the government, and there are only 0.59 caregivers per 100,000 population working in mental health facilities [22]. Today, lack of a mental health act, lack of mental health units, the absence of district-level mental health planning, inequitable allocation of funding for mental health, inadequate mental health record-keeping systems, very few health workers trained in mental health, and the unavailability of psychotropic drugs are limitations in the mental health system in Nepal [23]. Moreover, no local scientific evidence is available to support public health planning, implementation, and evaluation efforts. Thus, this study was conducted to estimate the presence and determine factors associated with depressive symptoms.

## Methods

### Study design and setting

This was an institutional-based cross-sectional study. The study was conducted in 22 CCHs of five districts of Nepal—Kathmandu, Lalitpur, Kaski, Chitwan, and Bhaktpur. The CCHs in these districts are operated by government and nongovernmental organizations. These districts were selected because they had a large number of CCHs and OVAs [16] that included adolescents from highly populated and diverse population areas from all over the country. In this way, adolescents representing various cultures, ethnicities, and economic statuses of the country would be included in the study.

### Study population

The study participants were 13–17-year-olds residing in CCHs; these adolescents were defined as OVAs in this study. OVAs include those who lost one or both parents or whose parents were in prison, those who ran away from home or were found living in public places and were very poor, those who had no proper rearing and care from their parents due to the physical and/or mental disability of parents, adolescents with HIV, and those staying in CCHs for any other reason [16]. According to the Children’s Act 2018, “children” refers to persons who have not reached 18 years of age. In this study, children aged 13 to 17 years were recruited. Persons of this age group are also referred to as adolescents.

### Sample size calculation and sampling technique

By the sample size calculation formula for a cross-sectional [24], at Z_α/2_ = 1.96, α = 0.05, *p* = 0.52 [25], and considering a 20.0% nonresponse rate, then at least 459 samples were required for the analysis. The five study districts were selected purposively, and the CCHs in the selected districts were selected by a simple random method. Adolescents aged 13–17 years who lived in the selected CCHs were eligible for the study. Overall, 22 CCHs were selected from a total of 454 centers in five selected districts.

### Research instruments

A validated questionnaire and the BDI-II [26] were used to collect information from the participants. The questionnaire consisted of five parts. In part one, six questions collected general information of the participants, e.g., sex, age, education, and ethnicity. In part two, four questions collected the parents’ status such as their life status, history of parental visits, presence of other family members or relatives, and information of the parents. In part three, two major questions related to alcohol and smoking use and behavior. In part four, three questions collected health information such as information related to health problems (congenital disorders, asthma, COPD (Chronic obstructive pulmonary disease), HIV) and the weight and height of the participants. In part five, four questions collected information related to abuse and social support.

The validity of the questionnaire was assessed by Item Objective Congruence (IOC) carried out by three external experts: a psychiatrist, an epidemiologist, and a public health specialist. Questions that scored less than 0.5 were deleted from the questionnaire, whereas those that scored 0.5–0.7 were revised as per the comments from the IOC committee before being included in the questionnaire. Questions that scored greater than 0.7 were included in the questionnaire without revision. Translation and back-translation methods was used to validate the questionnaire. In the current study, a pilot test was conducted among 30 participants from two CCHs (Jyoti Ko Ghar Nepal and Nepali Griha) to assesses feasibility, comprehension, and order of the questions. The Cronbach’s α was found at 0.79.

### Beck depression inventory-II (BDI-II)

The BDI-II was used to measure the level of depression and consists of 21 self-reported questions. It is one of the most widely used instruments for measuring the severity of depression. Each question has 4 possible choices that are a given score from 0 to 3. The total score is used to interpret the severity of depression: 0–13 indicates minimal depression, 14–19 indicates mild depression, 20–28 indicates moderate depression, and 29–63 indicates severe depression [26]. In this tool, the total highest possible score is 63, and the lowest possible score is zero. This tool is applicable to people between the ages of 13 and 80 [27]. The sensitivity, specificity, and reliability of the Nepali version of the BDI-II are 85.0, 86.0, and 8.0%, respectively [28].

In the current study, those with BDI-II scores indicating mild to severe depression were defined as having clinically relevant depressive symptoms while those with a BDI-II score indicating minimal depression were defined as not having clinically relevant depressive symptoms.

### Data collection procedures

Ethical approval to conduct the study was obtained from all relevant ethical committees (three committees). After this, all of the heads of the selected CCHs were contacted and briefly informed about the objective of the study, and an appointment was made to visit the CCHs. Essential information relating to the proceedings of the study was given to the person assisting with the data collection and to the eligible participants. Written consent was obtained from the caregivers of the centers and from the participating adolescents. The data were then collected via the self-administered questionnaire. Before collecting the data, all questions were explained to the participants; any confusion or questions that arose while the participants were filling out the questionnaire were addressed. At the same time, the weight and height of the participants were measured. To reduce instances of missing data, all completed questionnaires were double-checked before leaving the CCH.

### Statistical analysis

All gathered data was checked, compiled, coded, and entered into MS Excel and then transferred into IBM SPSS version 20 (SPSS, Chicago, IL) for the analysis. In the descriptive analysis, categorical variables were analyzed by frequency and percentage, while the continuous variables were described as the mean and standard deviation (SD) to present the general characteristics of the participants. Logistic regression was used to determine the factors associated with clinically relevant depressive symptoms, and the significance at α = 0.05 was ascertained in the univariate and multivariate analyses. When selecting the independent variables for the model, the “ENTER” mode was used. Before model interpretation (including the final model), the Cox-Snell pseudo R^2^, the Nagelkerke R^2^, and the Hosmer-Lemshow chi-square values were used to determine the fit of the model.

## Results

A total of 602 OVAs were recruited into the study from 22 CCHs. The majority of the participants were female (51.0%), and the mean age was 14.7 years (SD ± 1.38). The majority of the participants were orphans (55.1%); of these, both parents had died for 46.9%, the father had died for 33.7%, and the mother had died for 19.2%. Most participants were studying at the secondary level (91.2%) and belonged to the indigenous ethnic group (32.2%), followed by Dalit (24.9%). In general, the participants had lived at CCHs since an early age, with an average age of 6.9 years at entry into the center (Table 1).

**Table 1:** General characteristics of the participants

Characteristics	n	%
**Total**	**602**	**100.0**
**Sex**		
Male	295	49.0
Female	307	51.0
**Age** (years)		
< 15	268	46.6
≥ 15	334	55.4
**Education**		
Primary	40	6.6
Secondary	549	91.2
High school	13	2.2
**Ethnicity**		
Indigenous	194	32.2
Dalit	150	24.9
Chhetri	116	19.3
Brahmin	83	13.8
Others	59	9.8
**Age of the entrance** (years)		
< 5	152	25.2
5–10	327	54.3
11–15	120	19.9
> 15	3	0.5
**Duration of stay in CCH** (years)		
< 1	31	5.1
1–5	188	31.2
5–10	303	50.3
> 10	80	13.3
**Orphanhood status**		
No	270	44.9
Yes	332	55.1
*Parental orphan (father died)*	*156*	*46.9*
*Maternal orphan (mother died)*	*64*	*19.2*
*Double orphan (both parents died)*	*112*	*33.7*
**Knowing parents’ home**		
Yes	517	85.9
No	85	14.1
**Having relatives**		
Yes	509	84.6
No	93	15.4
**Cared by parents**		
Yes	467	77.6
No	135	22.4

Regarding health behaviors, 2.8% used alcohol, and 1.2% smoked. Fifty-five persons (9.1%) had abnormal weight, and 28.2% had some form of health problem. Of the total participants, 4.7% currently suffered from some form of violence or abuse in the CCH; 12.3% had felt neglected by a teacher; 13.5% had low social support; and 16.6% were bullied because they were OVAs (Table 2).

**Table 2:** Behaviors, health conditions and social-related factors of the participants

Characteristics	n	%
**Alcohol use**		
Yes	17	2.8
No	585	97.2
**Smoking**		
Yes	7	1.2
No	295	98.8
**Health problem** (Congenital disorders, asthma, COPD, HIV)		
Yes	170	28.2
No	432	71.8
**Nutritional status**		
Underweight	39	6.5
Normal	547	90.9
Overweight	16	2.7
**Abuse**		
No	574	95.3
Yes	28	4.7
*Physical*	*11*	*39.3*
*Mental*	*15*	*53.6*
*Sexual*	*2*	*7.1*
**Social support**		
Low	81	13.5
Good	521	86.5
**Neglect by teacher**		
Yes	74	12.3
No	528	87.7
**Bullied by friends**		
Yes	100	16.6
No	502	83.4

The overall presence of clinically relevant depressive symptoms among participants was 33.2%; 66.8% had minimal, 17.4% had mild, 11.3% had moderate, and 4.5% had severe depression.

In the univariable analysis, six variables were found to be associated with clinically relevant depressive symptoms: sex, alcohol use, health problems, social support, bullying, and neglect from a teacher. However, after controlling for age and education in the multivariable analysis, five variables were found to be associated with clinically relevant depressive symptoms: sex, alcohol use, health problems, social support, and bullying. Females were 1.96 times more likely to develop clinically relevant depressive symptoms than males (95% CI = 1.36–2.83). The adolescents who used alcohol were 3.42 times more likely to develop clinically relevant depressive symptoms than those who did not (95% CI = 1.16–10.12). Those who had a health problem were 2.00 times more likely to develop clinically relevant depressive symptoms n than those who did not (95% CI = 1.36–2.94). Those who had low social support were 1.81 times more likely to develop clinically relevant depressive symptoms than those who had high social support (95% CI = 1.08–3.03), and those who were bullied were 1.97 times more likely to develop clinically relevant depressive symptoms than those who were not (95% CI = 1.23–3.15) (Table 3).

**Table 3:** Univariable and multivariable analyses to identify factors associated with clinically relevant depressive symptoms among OVAs in CCHs in Nepal

Factor	Univariable analysis			Multivariable analysis		
	OR	95% CI	*p*-value	AOR	95% CI	*p*-value
**Sex**						
Male	1.00			1.00		
Female	1.62	1.15–2.28	0.006*	1.96	1.36–2.83	< 0.001*
**Age** (years)						
< 15	1.00					
≥ 15	0.85	0.60–1.19	0.359			
**Education**						
Primary	1.00					
Secondary	1.31	0.64–2.69	0.450			
High School	2.63	0.69–9.94	0.216			
**Ethnicity**						
Brahmin	1.00					
Indigenous	1.73	0.91–3.29	0.094			
Dalit	1.30	0.66–2.54	0.436			
Chhetri	1.16	0.57–2.33	0.674			
Others	1.09	0.51–2.30	0.815			
**Duration of stay in the child care center** (year)						
< 1	1.00					
1–5	1.22	0.51–2.89	0.652			
5–10	1.54	0.66–3.57	0.308			
> 10	1.72	0.68–4.34	0.247			
**Knowing parents’ home**						
Yes	1.00					
No	1.49	0.93–2.38	0.094			
**Orphanhood status**						
No	1.00					
Yes	0.90	0.64–1.27	0.566			
**Relatives**						
Yes	1.00					
No	0.99	0.62–1.58	0.980			
**Alcohol use**						
Yes	3.84	1.39–10.54	0.009*	3.42	1.16–10.12	0.022*
No	1.00			1.00		
**Smoking**						
Yes	5.12	0.90–26.66	0.052			
No	1.00					
**Health problem**						
Yes	2.15	1.49–3.11	< 0.001*	2.00	1.36–2.94	< 0.001*
No	1.00			1.00		
**Nutritional status**						
Underweight	2.41	0.46–12.52	0.294			
Normal	3.66	0.82–16.29	0.088			
Overweight	1.00					
**Abuse**						
Yes	1.00					
No	0.55	0.26–1.19	0.133			
**Neglect by teacher**						
Yes	1.85	1.13–3.03	0.014*			
No	1.00					
**Social support**						
Low	1.95	1.21–3.14	0.005*	1.81	1.08–3.03	0.023*
Good	1.00			1.00		
**Bullied by friends**						
Yes	2.23	1.44–3.45	< 0.001*	1.97	1.23–3.15	0.005*
No	1.00			1.00		

* Significance level at α = 0.05

## Discussion

The overall presence of depressive symptoms among OVAs aged 13–17 years living in CCHs in Nepal was 33.2%, with 15.8% having moderate-to-severe depression. Being female, drinking alcohol, presence of congenital diseases, low social support, and getting bullied were found to be factors significantly associated with depressive symptoms among this population.

The presence rate of depressive symptoms found in the current study was lower compared with the presences reported by studies among adolescents in Ethiopia (36.4%) [29], India (46.0–53.0%) [30], and the Gaza Strip 67.9% [12], which are also developing countries. In the developed country of Japan, 43.3% [31] of children in residential foster care were found to have depressive symptoms; however, a systematic review reported that the prevalence of the depressive disorder in children in the Japanese child welfare system was 11.0% [32]. The variations in the presences of depressive symptoms found in these studies can partly be explained by differences in sample size, study population, and different methods of assessment. Most of the studies [29, 30] considered only the orphan population, whereas the current study included all children or adolescents living in CCHs, which more accurately reflects the true situation in a Nepalese context. Moreover, there were variations in the criteria used to categorize depression and in the depression measurement tools. Some studies [12, 30] used screening instruments, whereas other studies used diagnostic instruments [29]. The study settings were also different among the countries, with socioeconomic status and cultural variations. These factors might be why there were differences in the presence of depressive symptoms among the studies. In addition, the current study found a higher rate of the presence of depressive symptoms than has been identified both globally and nationally among general children and adolescents [7]. This might be because the profile of OVAs living in CCHs may result in them being more vulnerable to developing mental health problems compared to children or adolescents living with their parents.

In our study, females were more likely to develop depressive symptoms than males. This result is supported by the findings from Egypt [5], Southwest Ethiopia [4], and Cameroon [33]. The internal behavior of girls, the way in which they respond to stress, the effect of the adolescence stage, and the effect of cultural discrimination could be possible causes of higher depression rates among females compared to males. Moeini et al. [34] reported that in the puberty stage, there are various possible psychological risk factors that lead to depression, particularly among girls. Schimelpfening [35] reported that the social and socialization role of females, gender differences, coping mechanisms, stressful life events, and hormonal changes in females were found to be risk factors of depression. However, Demoze et al. [29] reported that sex was not significantly associated with depression among orphans.

We also found that those who used alcohol were more likely to develop depressive symptoms than those who did not. This finding was supported by an international study in primary care [36] which showed alcohol use was associated with depression. Similarly, another study conducted in Australia [37] showed that those who consumed alcohol were more likely to develop psychological distress. A study conducted in Norway [38] revealed that depression in adolescents was associated with the onset of alcohol consumption and that alcohol increased the risk of depression. This might be because high levels of alcohol consumption cause negative effects in many aspects of life, and it can also harm the brain, causing depression [39].

In this study, those who had a health problem were more likely to develop depressive symptoms than those who did not. Similarly, a study from Ethiopia revealed that the presence of medical illness increased the risk of depression [4], and a study in Cameroon [33] reported that the presence of chronic diseases increased the risk of depression. Awaad et al. [40] also reported that adolescents with chronic heart disease were at increased risk of depression. This might be because health problems not only affect daily activities but also school attendance, which in turn affects the psychological well-being of children and adolescents. Moreover, health problems also increase stress levels, which is one of the risk factors for depression among adolescents [3]. The Mental Health Foundation reported that medical problems can change one’s lifestyle, increase feelings of sadness and fatigue, and some diseases cause changes in the brain that increase the risk of mental health problems [41].

Furthermore, in the current study, those adolescents who had low social support were more likely to develop depressive symptoms than those who had high social support. This result is analogous to the findings of a study conducted in Ethiopia [29]. Another study conducted in the UK reported that a lack of social support from friends and family was a significant predictor of increased depression among adolescents aged 13–19 years [42]. Similarly, the findings of a meta-analysis [43] showed that social support affects mental health, and improving social support in families and neighbors reduces mental health problems. A national survey of mental health and well-being conducted in Australia reported that a high connection of social support reduced the likelihood of depression [44]. The WHO confirmed [3] that social support is a precursor of well-being, and low social support and distressed relationships can cause psychological distress and depression. Cheng et al. [43] also reported that social support in children and adolescents helps them to cope with daily stressors by protecting against negative stressors and promoting positive mental health. Low levels of social support in adolescents make them feel discriminated against and increases frustration, it also acts as a stressor that leads to depression. Wang et al. [45] found that good social support helps to improve one’s self-esteem and lessens negative cognition, which helps to reduce depression in adolescents.

In this study, OVAs aged 13–17 years in Nepal who had been bullied were more likely to develop depressive symptoms than those who were not bullied. This finding is supported by other studies [46, 47] that reported that bullied children or adolescents were more likely to develop depression than those who were not. Adolescents who are bullied are more likely to experience frustration and suffer from abuse, and they are more likely to eventually develop depression [47]. Coggan et al. [48] reported that bullied children have lower self-esteem, increased emotional dysregulation, stress and hopelessness, and are more likely to attempt self-harm and suicide than others who are predisposed to depression. They are also more likely to be lonely and to want to avoid school. Hong et al. [49] reported that children and adolescents were at higher risk of anti-social behaviors such as problems at school, substance use, and aggressive behavior, which increase the chance of depression.

To the best of our knowledge, this study is the first of its kind in Nepal. Moreover, the study was conducted in districts containing a large number of OVAs from diverse populations from all over the country, representing many cultures, ethnicities, and economic backgrounds. However, the participants were only those who lived in child care homes, which limits the generalization to OVAs who live in the community. The tool used in the study to identify the depression which is based on self-report, the result might be presented the exact situation of depression among the OVAs. It needs health professionals who have been trained in the field to identify the magnitude of the problem. The study design was not intended to have the comparison group, then in the step of extracting the factors associated with depressive symptoms may need the confirmation from a stronger epidemiological study design in the future. In addition, the study did not assess other psychopathologies and exiting relevant treatment to depression problem, then it lacks the overall scenario of the problem in these particular populations in Nepal. Finally, the authors used BDI-II which is not the structural diagnosis interview to identify depressive disorder, therefore, the findings of the study may be mixed up with false positives leading to reducing the precision of the study.

## Conclusions

The overall presence of clinically relevant depressive symptoms among OVAs in CCHs in Nepal is high. There is an urgent need for regular psychological assessment of these adolescents through a well-developed system that should be carried out for the early detection and proper management of mental health problems. The adolescents must be protected from abuse, their risk behavior should be reduced, and a suitable environment for the positive development of adolescents should be provided, particularly for females and for those who have medical conditions. Interventions should be aimed to reduce alcohol use and to teach OVAs to avoid bullying others to reduce anti-social behaviors and, thereby, reduce depressive symptoms. The orphans and vulnerable adolescents bearing any form of health problems should be provided with a suitable environment for their positive growth. Mental health policies in Nepal should focus on adolescents, with special attention given to vulnerable groups. Moreover, national-level studies are further needed to identify and explore the main causes of depressive symptoms and possible interventions to help vulnerable adolescents develop into more stable individuals.

## Supplementary information

**Additional file 1.** Questionnaire for prevalence and associated factors of depression among orphans and vulnerable children in child care homes in Nepal.

## Data Availability

The datasets used and/or analyzed during the current study are available from the corresponding author on reasonable request.

## Abbreviations

AOR: Adjusted odds ratio
BDI: Beck depression inventory
CCH: Child care home
COPD: Chronic obstructive pulmonary disease
HIV: Human immunodeficiency virus
OR: Odds ratio
OVA: Orphans and vulnerable adolescents
SPSS: Statistical package for the social sciences
