# Supplementary material for: Depressive symptoms among orphans and vulnerable adolescents in childcare homes in Nepal: a cross-sectional study
Source: BMC Psychiatry. 2020 Sep 25;20:466. doi: 10.1186/s12888-020-02863-y (PMC7517808; doi:10.1186/s12888-020-02863-y)
Supplement: Supplementary file 1 — Additional file 1. Questionnaire for prevalence and associated factors of depression among orphans and vulnerable children in child care homes in Nepal. [file 12888_2020_2863_MOESM1_ESM.pdf]

Questionnaire for prevalence and associated factors of depression among orphans and vulnerable children in child care homes in Nepal

Please fill in the blanks and shade the box of answers where necessary

Identification Number…………………. **Date:…..../……/……**

Part I: Demographic information of children

1. Gender: ☐ Male ☐ Female
2. Age: …………………..years
3. Education: ……………class
4. Ethnicity: ☐ Brahmin ☐ Chhetri ☐ Indigenous

   ☐ Dalit ☐ Others………………..
5. Age at the entrance in child care home: ………………years
6. Length of stay in this child care home: ………… year…………….month

Part II: Family related questionnaires

7. Do you have any idea about your home?

   Yes ☐ No ☐

8. Do you have Parents?

   Both mother & Father ☐; only Mother ☐; only father ☐;

   none (mother and father) ☐; don't know ☐

9. Do you have any biological parents or relatives?

Yes ☐ No ☐

10. Does anyone come to meet you here?

Yes ☐ No ☐

If yes; who? (Mention the relation with them)..

Part III Behavioral Factors of children

11. Do you take alcohol currently?

Yes ☐ No ☐

If yes, at what age had you started? Age…………..years

If no, did you take alcohol in the past?

Yes ☐ No ☐

12. Do you currently smoke any tobacco products such as smoke or smokeless tobacco product?

Yes ☐ No ☐

If yes, at what age had you started? Age…………..years

Do you currently smoke tobacco products daily?

Yes ☐ No ☐

Part IV Physical health

13. Are you suffer from any physical health problems?

Yes ☐ No ☐

If yes, which disease? ....

Weight =……….kg

Height =………..inch

Part IV: Socio-environmental Factors

14. In this center, does any person abuses to you?

Yes ☐ No ☐

If yes; which type of abuse

Physical abuse☐ Mental abuse ☐ Sexual abuse ☐ Others ☐

15. In your school, does the teacher neglect you because of your living in the child care homes?

Yes ☐ No ☐

16. How do you feel, does society supports you?

Low ☐ Medium ☐ High ☐

17. In school your friend bullying to you?

Yes ☐ No ☐

Beck Depression Inventory 2nd edition (BDI II) tool

Circle in any one ◯

1. Sadness

0. I do not feel sad.

1. I feel sad much of the time.

2. I am sad all the time.

3. I am so sad or unhappy that I can't stand it.

2. Pessimism

0. I am not discouraged about my future.

1. I feel more discouraged about my future than I used to be.

2. I do not expect things to work out for me.

3. I feel my future is hopeless and will only get worse.

3. Past Failure

0. I do not feel like a failure.

1. I have failed more than I should have.

2. As I look back I see a lot of failures.

3. I feel I am a total failure as a person.

4. Loss of Pleasure

0. I get as much pleasure as I ever did from the things I enjoy.

1. I don't enjoy things as much as I used to.

2. I get very little pleasure from the things I used to enjoy.

3. I can't get any pleasure from the things I used to enjoy.

5. Guilty Feelings

0. I don't feel particularly guilty.

1. I feel guilty over many things I have done or should have done.

2. I feel guilty most of the time.

3. I feel guilty all the time.

6. Punishment Feelings

0. I don't feel I am being punished.

1. I feel I may be punished.

2. I expect to be punished.

3. I feel I am being punished.

7. Self-Dislike

0. I feel the same about myself as ever.

1. I have lost confidence in myself.

2. I am disappointed in myself.

3. I dislike myself.

8. Self-Criticalness

0. I don't criticize or blame myself more than usual.

1. I am more critical of myself than I used to be.

2. I criticize myself for all of my faults.

3. I blame myself for everything bad that happens.

9. Suicidal Thoughts or Wishes

0. I don’t have any thoughts of killing myself.

1. I have thoughts of killing myself, but I would not carry them out.

2. I would like to kill myself.

3. I would kill myself if I had the chance.

10. Crying

0. I don’t cry any more than I used to.

1. I cry more than I used to.

2. I cry over every little thing.

3. I feel like crying, but I can’t.

11. Agitation

0. I am no more restless or wound up than usual.

1. I feel more restless or wound up than usual.

2. I am so restless or agitated that it’s hard to stay still.

3. I am so restless or agitated that I have to keep moving or doing something.

12. Loss of Interest

0. I have not lost interest in other people or activities.

1. I am less interested in other people or things than before.

2. I have lost most of my interest in other people or things.

3. it’s hard to get interested in anything.

13. Indecisiveness

0. I make decisions about as well as ever.

1. I find it is more difficult to make decisions than usual.

2. I have much greater difficulty in making decisions than I used to.

3. I have trouble making any decisions.

14. Worthlessness

0. I do not feel I am worthless.

1. I don't consider myself as worthwhile and useful as I used to.

2. I feel more worthless as compare to other people.

3. I feel utterly worthless.

15. Loss of Energy

0. I have as much energy as ever.

1. I have less energy than I used to have.

2. I don't have enough energy to do very much.

3. I don't have enough energy to do anything.

16. Changes in Sleeping Pattern

0. I have not experienced any change in my sleeping pattern.

1. I sleep somewhat less than usual. –or– I sleep somewhat more than usual.

2. I sleep a lot less than usual. –or– I sleep a lot more than usual.

3. I sleep most of the day. –or– I wake up 1-2 hours early and can't get back to sleep.

17 Irritability

0. I am no more irritable than usual.

1. I am more irritable than usual.

2. I am much more irritable than usual.

3. I am irritable all the time.

18. Changes in Appetite

0. I have not experienced any change in my appetite.

1. My appetite is somewhat less than usual. usual.

2. My appetite is much less than usual.

3. I have no appetite at all.

19. Concentration Difficulty

0. I can concentrate as well as ever.

1. I can’t concentrate as well as usual.

2. It’s hard to keep my mind on anything for very long.

3. I find I can’t concentrate on anything.

20. Tiredness or Fatigue

0. I am no more tired or fatigued than usual.

1. I get more tired or fatigued more easily than usual.

2. I am too tired or fatigued to do a lot of the things I used to do.

3. I am too tired or fatigued to do most of the things I used to do.

21. Loss of Interest in Sex

0. I have not noticed any recent change in my interest in sex.

1. I am less interested in sex than I used to be.

2. I am much less interested in sex now.

3. I have lost interest in sex completely.

Total __________ Score
